# Food safety assessment of crops engineered with RNA interference and other methods to modulate expression of endogenous and plant pest genes

**DOI:** 10.1002/ps.5957

**Published:** 2020-07-02

**Authors:** Gijs A Kleter

**Affiliations:** ^1^ Wageningen Food Safety Research Wageningen University and Research Wageningen The Netherlands

**Keywords:** RNA interference, crop biotechnology, gene editing, food safety, risk assessment, host‐induced gene silencing

## Abstract

Genetically modified crops have been grown commercially for more than two decades. Some of these crops have been modified with genetic constructs that induce gene silencing through RNA interference (RNAi). The targets for this silencing action are genes, either specific endogenous ones of the host plant or those of particular pests or pathogens infesting these plants. Recently emerging new genetic tools enable precise DNA edits with the same silencing effect and have also increased our knowledge and insights into the mechanisms of RNAi. For the assessment of the safety of foodstuffs from crops modified with RNAi, internationally harmonized principles for risk assessment of foods derived from genetically modified crops can be followed. Special considerations may apply to the newly expressed silencing RNA molecules, such as their possible uptake by consumers and interference with expression of host genes, which, however, would need to overcome many barriers. Bioinformatics tools aid the prediction of possible interference by a given RNA molecule with the expression of genes with homologous sequences in the host crop and in other organisms, or possible off‐target edits in gene‐edited crops. © 2020 The Author. *Pest Management Science* published by John Wiley & Sons Ltd on behalf of Society of Chemical Industry.

## Graphical abstract

RNA interference can confer pest resistance and alter the nutrient composition of genetically modified crops. Their food safety assessment should follow a harmonized approach, supported by bioinformatics and omics techniques.

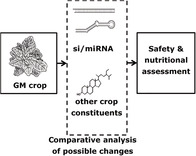

## 1 INTRODUCTION

Since the mid‐1990s, there has been a rapid adoption and expansion of genetically modified (GM) varieties of major commodity crops by farmers in many parts of the world, accruing to 192 million hectares in 2018.^1^ A major part of these crops is accounted for by a limited number of crop species in which specific, newly expressed proteins are produced, imparting traits of herbicide tolerance and insect resistance. Herbicide tolerance, for example, can be achieved through the expression of enzymes that either (i) convert the phytotoxic herbicide active ingredient, such as glyphosate or glufosinate, to less toxic metabolites, or (ii) are inhibition‐resistant analogues of intrinsic crop enzymes, such as 5‐enolpyruvylshikimate‐3‐phosphate synthase (EPSPS) or acetolactate synthase (ALS), which are targeted and inhibited by the active ingredient. Herbicide‐tolerant crops are able to withstand the otherwise toxic action of the herbicide and thereby sustain its action when it is applied with broadcast sprays over the top of crops plants within a farm field, while still eliminating the weeds between the crop rows. In insect‐resistant crops, an insecticidal protein is commonly expressed at low levels, such as Cry and VIP proteins. Cry proteins occur naturally in the endosporal crystal inclusions of *Bacillus thuringiensis*, whilst VIP proteins are formed during the vegetative stages of this bacterium. Proteins from both categories are selectively toxic for particular insect species, such as larvae of lepidopterans (moths) and coleopterans (beetles) feeding on these crops.

Besides these crops expressing proteins encoded by the introduced genes, repression of protein expression is actually the target of the genetic modifications in various other commercial and precommercial GM crops. This mechanism, commonly designated gene silencing, was particularly used in virus‐resistant papaya and cucurbits in the early days of GM crop commercialization as well as oilseed and starch crops with modified fatty acid and starch profiles, respectively. Although it was known then that the introduction of sense and antisense DNA homologues of endogenous genes could bring about this gene silencing, knowledge of the underlying mechanism, called RNA interference (RNAi), has been accruing significantly since. This has also led to insights that triggered further advancement of this technology.

## 2 RNAI IN CROP BIOTECHNOLOGY

Examples of gene‐silenced GM crops in which RNAi is used for pest control include virus‐resistant crops expressing sense/antisense constructs. Various crops, for example, have been modified with sequences of virus coat proteins of invading viruses, such as papaya that has thus been rendered resistant against the papaya ringspot virus, potatoes against potato leaf roll virus or potato virus Y, and cucurbits against cucumber mosaic virus or zucchini yellowing mosaic virus.^2^ Hence in these cases it is the genes of the invading pathogen or infesting pest that are suppressed, preventing their further spread to other plants or even eliminating them. A recent addition to the range of commercial GM crops exerting RNAi in pests is GM maize expressing double‐stranded RNA (dsRNA; with a hairpin loop) targeting larvae of the Western corn rootworm. This insect belongs to the order of Coleoptera, which are known to be particularly sensitive towards the action of dsRNA administered orally, unlike others, such as Lepidoptera, which may be less sensitive.^3^

As mentioned above, RNAi has also been exploited by genetic engineers to alter nutrient composition in edible crop parts, such as seeds or tubers. This is achieved by knocking out the expression of endogenous genes involved in nutrient formation, such as for fatty acid dehydrogenation or amylose starch formation. Examples include various GM oilseed crops with increased levels of the monounsaturated fatty acid oleic acid (C18:1). This increased level of a monounsaturated fatty acid at the expense of polyunsaturated fatty acids such as linoleic (C18:2) and linolenic (C18:3) acids has been achieved through silencing further biosynthetic steps from monounsaturated towards polyunsaturated acids, including, for example, the use of constructs silencing the expression of genes coding for fatty acid dehydrogenase enzymes. In addition, the silencing of enzymes involved in the biosynthesis of amylose, i.e. the enzyme granule‐bound starch synthase (GBSS), affords the production of amylose‐free starch crops such as starch potato. In this way, no amylose needs to be separated first using harsh chemical procedures from the amylopectin starch before the latter can be further used in industrial starch potato processing.

## 3 RECENTLY EMERGING TECHNOLOGIES FOR GENE ACTIVITY ALTERATION

RNAi has traditionally been an important strategy employed by genetic engineers for gene silencing in plants. A variant of RNAi which has recently entered the precommercialization regulatory track is that of RNA‐dependent RNA methylation. Various precommercial gene‐edited crops in which such methylation has been achieved as an epigenetic effect have successfully passed the US Department of Agricultureʼs Am I regulated procedure, in which petitioners receive its view on whether or not these crops are to be considered genetically engineered varieties.^4^

The advent of new plant breeding techniques, including precise, low‐key molecular tools such as CRISPR Cas9 and other so‐called site‐directed nuclease enzymes, is set to revolutionize crop breeding. These techniques may also allow for more refined, targeted, and efficient mutation or blockage of target genes that could lead to their inactivation or inhibition of their expression.

## 4 THE MECHANISM OF RNAI AND GENE INACTIVATION USING SITE‐DIRECTED NUCLEASES

Over the past few decades, knowledge has been accruing on the various natural mechanisms through which plants modulate the activity of endogenous genes. A well‐investigated example is RNA interference (RNAi) based on the inhibiting activity of small RNA molecules of 20–24 nucleotides (nt) in length on the expression of corresponding genes at the transcriptional and post‐transcriptional stages. In addition, it recently has become known that small RNA molecules may also interfere with mRNA splicing, hence directing also the resulting mRNA formed post slicing and available for translation into proteins. It is thought that such RNAi mechanisms allow plants to rapidly modulate gene expression when this is needed, such as under stressful conditions.^5^ Besides this, RNAi also serves as a host defense against plant viruses and pathogens, as well as an avenue for interaction with symbiotic microorganisms.

**Figure 1:**
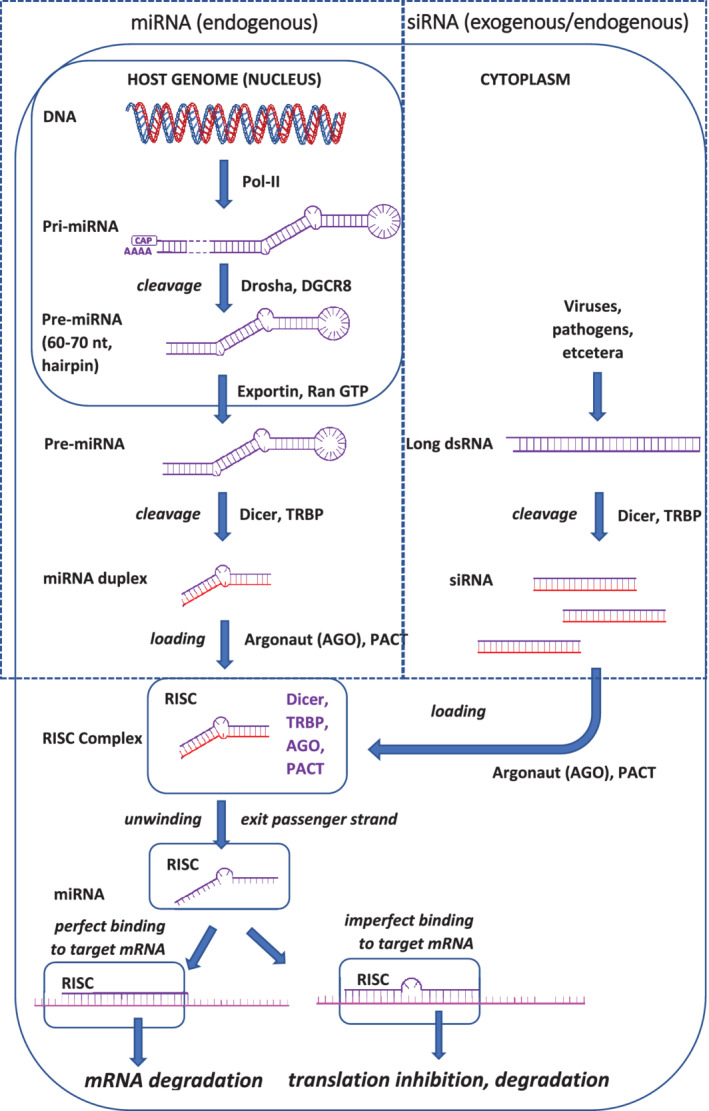
Schematic representation of miRNA and siRNA pathways towards RNAi‐based gene silencing in plant cells.

The small RNAs (sRNAs) involved with these pathways can be discerned as micro‐RNAs (miRNAs) formed from hairpin structures of single RNA molecules, and various kinds of small interfering RNA (siRNA), which originate from double‐stranded RNA molecules (such as certain RNA viruses), but also other sRNAs. A difference between miRNA and siRNA is that the specificity of siRNA is higher, requiring full alignment of a single strand of siRNA with complementary mRNA. By contrast, miRNA molecules can bind to multiple targets, aligning with the 3′ untranslated regions of mRNA. A simplified depiction of these various pathways and how they come together in eukaryotic cells is provided in Fig. 1. These different mechanisms share a common pathway through which the host plant degrades RNA complexed with the miRNA and siRNA. Components of this pathway are Dicer, Argonaut and the RNA‐induced silencing (RISC) complex as well as the guide strand of RNA. The Dicer enzyme is a ribonuclease enzyme, degrading pre‐miRNA or dsRNA into shorter fragments of miRNA and siRNA, respectively. From these double‐stranded fragments, the most stable strand (guide strand) is recognized and bound by Argonaut whilst degrading the other, passenger strand. Argonaut, Dicer and the guide RNA strand plus various other components then form the RISC complex. This complex will recognize complementary RNA, such as mRNA, of which the binding through Watson–Crick base‐pairing will trigger its degradation or modification. Many different types of RNA are recognized and trimmed by Dicer, leading to a wide array of different categories of derived products with varying functions.^6^, ^7^ This evolving knowledge on the various forms and functions of RNA, in turn, also bears the prospect of exploiting these mechanisms to modify plant nutrient composition in new GM crops or by using externally applied RNA‐based effectors, for example.

As mentioned above, CRISPR Cas9 and other site‐directed nucleases can also be used to inactivate genes. These enzymes act through the formation of double‐breaks in the targeted DNA at specific locations and the subsequent reannealing of the broken DNA through nonhomologous end‐joining (NHEJ). Specific base mutations can be achieved if a template oligonucleotide is used in the process. This way, the formation of indels (small insertions and deletions) and larger deletions may cause amber or frameshift mutations within the coding sequence, possibly leading to knock‐out of the target gene. Besides this, CRISPR interference is a new technology that employs the combination of a catalytically inactive Cas9 enzyme (dCas9) together with an sgRNA strand that directs it to the target DNA sequence. Binding of Cas9 to the latter may then cause inhibition of transcription initiation or elongation through steric hindrance of the RNA polymerase, depending on the binding position on the DNA.^8^ In addition, the use of chimeric proteins consisting of the inactive dCas9 fused with transcriptional regulator domains, in combination with sgRNA strands directing the dCas9 to promoter regions, can be used to repress or activate the activity of genes controlled by these promoters.^9^

## 5 SAFETY ASSESSMENT STRATEGY

### 5.1 The harmonized approach for GM crops

The safety assessment strategy for RNAi‐derived products generally follows that for a new GM crop variety. The latter is performed according to an internationally harmonized approach, which is laid out in guidelines for the safety assessment of plants created with recombinant DNA methods published by Codex Alimentarius in 2003.^10^ Codex Alimentarius is a joint, standard‐setting collaboration on food quality and safety between the Food and Agriculture Organization of the United Nations and the World Health Organization. Central to the harmonized approach is the comparative analysis of a GM crop and a conventional comparator with a history of safe use. This usually entails a molecular characterization of the inserted genetic material as well as an extensive analysis of the compositional, agronomic and phenotypic characteristics of the GM crop and its counterparts grown in field trials in a range of locations. This comparison will reveal both intended and possibly unintended changes to the GM crop. Based on the differences identified, a decision can be made which further studies are needed to assess the safety. This usually entails studies on the possible toxicity and allergenicity of any newly expressed proteins, which is studied according to a weight of evidence approach employing different methods, such as bioinformatics, *in vitro* and, optionally, *in vivo* studies on the protein. If other compositional parameters are changed, this may also trigger assessment of their safety and nutritional impact based on existing knowledge and the natural variation in crop varieties known to be safe, as well as further testing, in exceptional cases, with these compounds in purified form.

**Figure 2:**
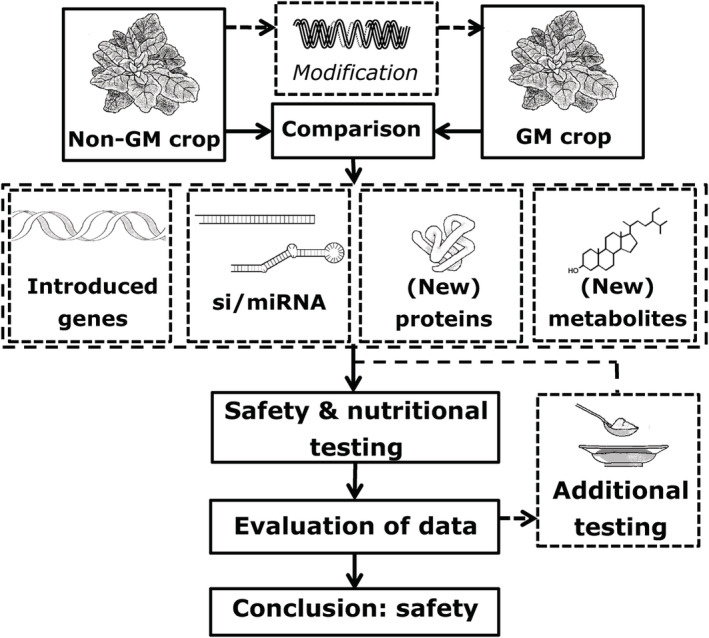
Comparative safety assessment approach for GM crops modified with RNA interference.

For RNAi‐modified GM crops, it can be envisaged that special conditions prevail. For example, the modification is not aimed at the expression of newly introduced proteins but rather to produce particular forms of dsRNA. Hence for the RNAi crop, the safety assessment will share a number of features, with a few exceptions, with those for other GM crops. Besides the characteristics of the parent crop, the donor, transgene and delivery process, the characteristics of the gene products (such as dsRNA) and of the new, transformed crop (such as its composition, agronomic and phenotypic traits) are essential data elements for a safety assessment, whilst recognizing that certain elements may not have been expressed due to RNAi (Fig. 2). In addition to the intended effects of the genetic modification process, the assessment will furthermore be focused on the potential occurrence of unintended effects caused by it.

The RNAi‐based crops that have been marketed so far often contain either stacked events bringing together different inserted, transgenic DNA constructs, or single constructs with multiple foreign genes. These other genes code for, for example, additional insecticidal toxins against the same insect pest targeted by a given dsRNA (enhancing effectiveness and reducing the likelihood of pest resistance development), herbicide resistance, or silencing genes directed against additional plant viruses. Obviously, these transgenic components will also have to be assessed for their safety and approved before they can be marketed.

### 5.2 Safety of silencing RNA and gene‐editing applied to crops

Much work has been done on the efficiency of RNAi in insect and nematode pests, in which also the conditions that prevail within their guts (e.g. nucleases), the existence of cellular surface receptors/membrane channels (e.g. Sid proteins) facilitating the systemic uptake from the gut, the interaction with the hostʼs RNAi machinery, and the corollary presence of viruses suppressing or saturating the hostʼs RNAi machinery have been investigated.^11^ These conditions in insects, which are conducive to dsRNA toxicity, generally do not apply to humans and food‐producing animals, except for crustaceans and molluscs, for which limited data available indicate sensitivity of several of the tested species.^12^

As the dsRNA may target sequences of genes that also have their analogs in humans and production animals, this raises questions regarding the theoretically possible interference of orally ingested dsRNA with the consumerʼs intrinsic mRNA.

The safety of dsRNA ingested with food has been extensively reviewed previously by various authors, describing the hazard(s) of dsRNA, siRNAs, and miRNAs for human/animal health.^13^, ^14^ They conclude that there is no evidence for adverse effects, whilst the oral uptake is low, and there are many gastrointestinal tract barriers. Furthermore there is a history of consumption of RNA as part of foods without known adverse effects *per se*. This finding of a lack of gut absorption and adverse effects of dietary dsRNA was further supported by a proof of concept 28‐day oral toxicity study. For this study, dsRNAs were used that would target vacuolar ATPase (vATPase). Whilst the suppression of this gene is effective in controlling corn rootworms,^3^ the material tested in this study had been designed to have 100% sequence identity to the mouse orthologue. A longer dsRNA (218 bp) or a pool of four 21‐mer vATPase siRNAs elicited no adverse effects in mice after 28 days of exposure at vastly greater levels than present in a normal diet (up to 64 mg kg^–1^ diet). No meaningful differences were noted in the level of vATPase mRNA expression in the brain, liver, kidney, stomach, duodenum, ileum, and spleen of the exposed mice.^15^ Similar findings were reached for dsRNA targeting DvSnf7 in Western corn rootworm, tested in mice.^16^

The US Environmental Protection Agency's (EPA) Scientific Advisory Panel (SAP) has considered the safety issues that may be particularly at stake for dsRNA, both as plant‐incorporated protectants (PIPs) expressed in GM plants, and as externally applied agents (pesticides, resistance factor repressors, developmental disruptors, growth enhancers). In its problem formulation published in 2014, the Panel advises, for example, to test for potential targets of silencing RNA using bioinformatics. Whilst dsRNA was generally known to be rapidly degraded in the gastrointestinal tract, it still identified a need for such information on PIPs, including their stability, potential uptake from the gut, and tissue distribution in consumers. It was also advised to consider dermal and inhalatory exposure besides oral exposure, although this might be more relevant to ectopically applied dsRNA, such as sprayable formulations applied to crops, than for PIPs.^17^

In its assessment of MON87411 maize expressing *DvSnf*7 dsRNA in 2016 (as part of a stacked maize event), EPA SAP also considered possible human health impacts. It discussed, for example, the improbability that the *DvSnf7* dsRNA in MON87411 (with 240 nt stem and 150 nt loop structure) would be able to form viroids. Moreover, it assessed the evidence for a potential uptake of exogenous dsRNA from the gut and its interference with host cell functions, for which there appeared to be neither reliable nor consistent indications. It also contended that bioinformatics outcomes indicating matches between dsRNA and human counterparts do not yet signify risks in the absence of abundance and functionally relevant exposure. In addition, there are a number of barriers that need to be overcome before exogenous RNA could reach its cellular targets and exert any effects, such as clearance mechanisms, cellular barriers, and high stoichiometric amounts to load intracellular Argonaut complexes of the RNAi machinery.^18^ This also concurs with lessons from the challenges faced by the development of RNAi‐based therapeutics, for which stabilization of RNA (e.g. through chemical modification), facilitation of uptake, and persistence within the body (e.g. liver) are targeted in order to attain relatively high doses of an RNA molecule of interest within humans and animals. MON87411 maize has also been assessed by other US agencies (USDA, FDA) in parallel, as well as by a swathe of foreign countries, including but not limited to the EFSA GMO Panel within the EU.

Interestingly, the bioinformatics‐supported analysis of possible effects of RNAi in humans and animals is aided by a range of open‐source and commercial predictive tools. These tools have been developed to assist researchers and product developers with designing their RNAi experiments and products, respectively. This way, the efficiency of the miRNA sequence to be used can be improved and potential unwanted off‐target effects avoided. These tools include databases with data on possible targets in, for example, plants, humans, and animals based on miRNAs that have already been identified or their transcriptomes. In addition, predictive algorithms are available, aligning sequences of interest to possible off‐target sequences, taking into account various rules for alignment, such as the presence of seed sequences, miRNA response elements (MREs), and a minimum level of homology required. The latter also implies that matches need not always be perfect but that mismatches to a certain extent are tolerated.^19^

Whereas siRNA are considered to be more specific than miRNA as they require full alignment with mRNA, off‐target effects may still occur if they behave as miRNA, aligning with 3′ untranslated mRNA regions. Another, nonspecific potential off‐target effect described in literature is saturation of the cellular RNAi machinery by high intracellular levels of dsRNA introduced either through transgene expression or administration of dsRNA. This way, endogenous miRNA cannot be properly processed anymore,^20^ yet this scenario appears not realistic for plant genetic improvement and the action of RNA molecules ingested with food.

A recent literature review carried out for the EFSA GMO Panel on the food and feed safety of exogenous, noncoding RNAs focused on three particular safety items: impact on gastrointestinal tract, systemic effects, and impacts on the immune system.^21^ This review supplemented two previous ones on molecular characterization and on environmental risks. Moreover, these reports had been preceded by an international scientific workshop with >100 participants hosted by EFSA on the risk assessment of RNAi‐based crops in 2014. Based on the extensive elaboration of food safety data found, it was concluded, amongst other things, that any RNA within the food would face many biological and physical and barriers to be overcome, such as RNA‐degrading ribonuclease enzymes, to be taken up, transported to other tissues and achieve effect intracellularly. Whilst there is little information on the impact of small exogenous noncoding RNAs on intestinal and adjoining tissues and organs, the fraction of surviving RNA is already considered to be very low, unless some form of stabilization is applied. Moreover, the molecules will need to escape degradation following uptake by endosomes or to overcome barriers between different body compartments, for example. Previous reports on the low presence of noncoding RNAs from plant foods in physiological fluids have to be viewed critically as there is potential confounding with technical artefacts and contamination. The systemic effect of orally ingested plant‐derived noncoding RNAs has not been unequivocally established as there is conflicting evidence for this, whilst it is still unclear if certain conditions, such as diet, could promote transfer of RNA. The report also identified data needs for the impact of small, plant‐derived RNA molecules on immune function, both directly on immune cell functioning and via effects on the gut microbiota.^21^

Contrary to gene‐silenced crops based on RNAi technology, there has so far been very limited experience with the commercialization of gene‐edited crops with silenced genes, neither have any guidelines for the food safety assessment of such crops been published yet. The reportedly first commercial crop of this kind was the high‐oleic soybean branded Calyno® from the Calyxt company. In February 2019, this company completed its consultation on Calyno® with the US FDA.^22^ Frying oil produced from this soybean is reportedly sold to and used in US restaurants. Using Transcription Activator‐Like Effector Nuclease (TALEN) molecular scissors, genetic engineers removed small (63‐ and 23‐bp) DNA fragments from the soybean *FAD2‐1A* and *FAD2‐1B* genes, respectively. These genes encode fatty acyl dehydrogenase enzymes that are involved in the conversion of the monounsaturated fatty acid oleic acid (C18:1) into the polyunsaturated fatty acid linoleic acid (C18:2). Consequently, the fatty acid profile of the Calyno® soybean compared to its control showed a preponderance of oleic acid, whereas the levels of, amongst others, linoleic acid and linolenic acid (C18:3) had decreased correspondingly. Besides the fatty acid profile, other compositional data had also been provided to the FDA to check for unintended changes in these components. These included a nutrient analysis comprising lecithins, proximates, amino acids, isoflavones, and various antinutrients (e.g. lectins, phytate, trypsin inhibitor, stachyose, raffinose). The fatty acid profile was compared to that of other high‐oleic oils that are already on the market, whilst reference was also made to previously assessed transgenic, RNAi‐based high‐oleic soybeans (from different companies). The nutrient composition (besides fatty acids) of Calyno® was stated to be similar to its comparators. These data provided in support of Calyno®ʼs safety are not different from the extensive compositional analysis that is commonly performed for GM crops, verifying both the intended and possible unintended changes caused by the genetic modification. This also holds true for the compositional premarket assessments of commercial herbicide‐tolerant crops with single‐nucleotide mutations assessed by the Canadian authorities as ‘plants with novel traits’, such as sulfonylurea‐herbicide‐tolerant oilseed rape event 5715 currently sold in Northern America by Cibus under the tradename Falco®.^23^

## 6 METHODS FOR THE PREDICTION AND DETECTION OF UNINTENDED EFFECTS OF RNAI‐MODIFIED PLANTS AND OFF‐TARGET EFFECTS OF GENE‐EDITING IN PLANTS

For the prediction of off‐target effects of the RNAi within the host plant, bioinformatics‐supported approaches can be followed. This is to identify homologies of the RNA to be expressed with intrinsic genes based on genomic data available on the host. Some of the databases and algorithms available include potential targets in plant species.^19^ Obviously, such tools may already be employed by developers of RNAi crops when designing the DNA constructs to modify the host crop, so as to avoid undesirable off‐target effects.

Notably, the EFSA GMO Panel published an internal note with recommendations on how to perform bioinformatics for the identification of potential off‐target genes as part of the safety assessment of RNAi‐based GM plants. It provides details on how many mismatches and gaps are allowed, for example, in the alignment of the sequence of the silencing RNA (miRNA, siRNA) with other RNA transcripts from the host plant as stored in transcript sequence databases. An alignment complying with these criteria can be considered a ‘hit’. Whereas transcripts with multiple hits should be the prime focus of the evaluation of potential misregulation by off‐target interactions, those with single hits may also be interesting in case high levels of the newly expressed silencing RNA molecule are produced by the modified host plant. The occurrence or absence of possibly related changes in the composition and phenotype of the modified crop will aid the interpretation of the relevance of the hits thus found.^24^

Bioinformatics tools are also available for predicting off‐target mutations caused by gene editing. For CRISPR Cas9, for example, the OFFinder tool aids the identification of potential off‐target sites where the nuclease may also have created double‐stranded DNA cuts that are prone to error when repaired, with, for example, insertions, deletions, and point mutations.^25^

Compositional analysis may be performed by targeted analysis of single compounds also including analytes beyond the common set of key nutrients, antinutrients, and toxins, as suggested by OECD consensus documents for particular crop species.^26^ These additional analytes should be selected based on their role within a broad spectrum of relevant pathways. Whereas ‘omics’, such as transcriptomics, might be applied to analyse for potential off‐target effects,^18^ it should be realized that such omics are not routinely applied in risk assessment. Moreover, the large datasets they produce pose challenges for their interpretation. To this end, the one‐class classification model, which is a statistical model, helps to define the acceptable boundaries of the known classes of commercial varieties considered as safe, for example. If profiles are classified outside this class, further research, or analysis of available data, may be needed, but this does not necessarily imply that they are unsafe.^27^

## 7 CONCLUSION

Gene‐silenced GM crops have been around from the early days of agricultural crop biotechnology. Nonetheless, a surge in commercial applications in food and feed production can be expected in the near future owing to our increased knowledge about RNA interference, the accessibility of tools for directed crop mutagenesis, and the expanding scope of experimental applications. This also begs the question if the safety assessment approaches that have been hitherto followed for GM crops are still sufficiently adapted. Data suggest that dsRNA ingested as consumed crop components does not raise particular issues with regard to off‐target effects in humans and animals given the history of food in which RNA naturally occurs in general, and the many barriers to overcome in order to have an effect. Bioinformatics have a key role in the prediction of possible unintended, off‐target effects of the dsRNA in the crop host itself, which could lead to alterations of, for example, crop composition, and indirectly introduce hazards for safety and nutrition. Omics approaches could help to characterize an RNAi‐modified plant in a holistic manner, whilst appropriate statistical tools may help to interpret the outcomes of such complex analyses in terms of similarity of this plant to varieties considered safe.
